# 301. Detection of Pneumococcal Pneumonia During SARS-CoV-2 Infection

**DOI:** 10.1093/ofid/ofab466.503

**Published:** 2021-12-04

**Authors:** Anne Watkins, Devyn Yolda-Carr, Isabel M Ott, Maura Nakahata, Adam Moore, M Catherine Muenker, Maria Tokuyama, Chantal B Vogels, Melissa Campbell, Rupak Datta, Charles Dela Cruz, Shelli F Farhadian, Akiko Iwasaki, Albert I Ko, Nathan D Grubaugh, Ronika Alexander-Parrish, Adriano Arguedas, Bradford D Gessner, Daniel Weinberger, Anne Wyllie

**Affiliations:** 1 Yale School of Public Health, New Haven, Connecticut; 2 UBC, Vancouver, British Columbia, Canada; 3 Yale School of Medicine, New Haven, Connecticut; 4 Yale School of Medicine - Yale New Haven Hospital, West Haven, CT; 5 Pfizer, Inc, Bowie, MD; 6 Pfizer Inc, Collegeville, Pennsylvania; 7 Pfizer Vaccines, Collegeville, PA

## Abstract

**Background:**

*Streptococcus pneumoniae* (pneumococcus) is a common colonizer of the upper respiratory tract and can progress to cause invasive and mucosal disease. Additionally, infection with pneumococcus can complicate respiratory viral infections (influenza, respiratory syncytial virus, etc.) by exacerbating the initial disease. Limited data exist describing the potential relationship of SARS-CoV-2 infection with pneumococcus and the role of co-infection in influencing COVID-19 severity.

**Methods:**

Inpatients and healthcare workers testing positive for SARS-CoV-2 during March-August 2020 were tested for pneumococcus through culture-enrichment of saliva followed by RT-qPCR (to identify carriage) and for inpatients only, serotype-specific urine antigen detection (UAD) assays (to identify pneumococcal pneumonia). A multinomial multivariate regression model was used to examine the relationship between pneumococcal detection and COVID-19 severity.

**Results:**

Among the 126 subjects who tested positive for SARS-CoV-2, the median age was 62 years; 54.9% of subjects were male; 88.89% were inpatients; 23.5% had an ICU stay; and 13.5% died. Pneumococcus was detected in 17 subjects (13.5%) by any method, including 5 subjects (4.0%) by RT-qPCR and 12 subjects (13.6%) by UAD. Little to no bacterial growth was observed on 21/235 culture plates. Detection by UAD was associated with both moderate and severe COVID-19 disease while RT-qPCR detection in saliva was not associated with severity. None of the 12 individuals who were UAD-positive died.

**Conclusion:**

Pneumococcal pneumonia (as determined by UAD) continues to occur during the ongoing pandemic and may be associated with more serious COVID-19 outcomes. Detection of pneumococcal carriage may be masked by high levels of antibiotic use. Future studies should better characterize the relationship between pneumococcus and SARS-CoV-2 across all disease severity levels.

**Disclosures:**

**Akiko Iwasaki, PhD**, **4Bio** (Consultant, Advisor or Review Panel member)**Adaptive Biotechnologies** (Consultant, Advisor or Review Panel member)**Blavatnik** (Grant/Research Support)**HHMI** (Grant/Research Support)**Mathers** (Grant/Research Support)**NIH** (Grant/Research Support)**Spring Discovery** (Grant/Research Support)**Spring Discovery** (Consultant, Advisor or Review Panel member)**Vedanta InProTher** (Consultant, Advisor or Review Panel member)**Yale School of Medicine** (Grant/Research Support) **Nathan D. Grubaugh, PhD**, **Tempus Labs** (Consultant) **Ronika Alexander-Parrish, RN, MAEd**, **Pfizer** (Employee, Shareholder) **Adriano Arguedas, MD**, **Pfizer** (Employee) **Bradford D. Gessner, MD, MPH**, **Pfizer Inc.** (Employee) **Daniel Weinberger, PhD**, **Affinivax** (Consultant)**Merck** (Consultant, Grant/Research Support)**Pfizer** (Consultant, Grant/Research Support) **Anne Wyllie, PhD**, **Global Diagnostic Systems** (Consultant)**Pfizer** (Advisor or Review Panel member, Research Grant or Support)**PPS Health** (Consultant)**Tempus Labs, Inc** (Research Grant or Support)

